# Magnesiophilic Interface of 3D MoSe_2_ for Reduced Mg Anode Overpotential

**DOI:** 10.3389/fchem.2020.00459

**Published:** 2020-06-18

**Authors:** Tong Shen, Chengzhao Luo, Yu Hao, Yu Chen

**Affiliations:** ^1^School of Optoelectronic Science and Engineering & Collaborative Innovation Center of Suzhou Nano Science and Technology, Soochow University, Suzhou, China; ^2^National University of Singapore Suzhou Research Institute, Dushu Lake Science and Education Innovation District, Suzhou, China

**Keywords:** magnesiophilic interface, Magnesium battery anode, MoSe_2_, low voltage hysteresis, porous structure

## Abstract

A large overpotential is often reported for rechargeable magnesium batteries during the deposition/stripping of magnesium, which can be detrimental to the cell performance. In this work, a three-dimensional electrode that mainly composed magnesiophilic MoSe_2_ (MMSE) has been fabricated and proposed as the substrate for the electrochemical deposition/stripping of magnesium metal. The magnesiophilic interface of MoSe_2_ has been proven by electrochemical tests of magnesium deposition test. In addition, the electrochemical property of 3D MMSE has been examined by a large-capacity (10 mAh/cm^2^) magnesium deposition/stripping test. The stable magnesiophilic interface of MMSE has been further confirmed by SEM characterization. Finally, the crucial effect of the magnesiophilic interface of MMSE on the overpotentials have been demonstrated by Mg deposition/stripping test under various current densities.

## Introduction

Strong interest in alternative energy storage systems has been caused due to the cost constraints and operational safety issues of lithium-ion batteries. Rechargeable magnesium battery is considered to be one of the most promising alternatives due to the low electrode potential (−2.36 V vs. NHE) and high-volume specific capacity (3,833 mA h cm^−3^) of Mg metal (Yoo et al., 2013; Mohtadi and Mizuno, 2014; Muldoon et al., 2014). The most fascinating feature of Mg metal anode is that there is no dendrite generation during the magnesium charge and discharge process, which eliminates the potential safety hazard of the battery short circuit caused by dendrite puncturing of the separator. However, the development of magnesium rechargeable battery is still limited by multiple obstacles, including the slow diffusion of highly polar divalent magnesium ions in the cathode material, the narrow voltage window of the electrolyte limiting the requirements of high energy density, the compatibility of the electrolyte with the magnesium metal anode, and the huge overpotential of both cathode and anode (Cheng et al., 2016; Canepa et al., 2017). Extensive effort has been devoted in the performance enhancement of rechargeable magnesium batteries. However, there are few studies focusing on the reduction of Mg anode overpotential (Li et al., 2018; Tang et al., 2019).

Electrode polarization can be categorized into electrochemical polarization, concentration polarization, and ohmic polarization. In magnesium battery, due to the bivalency of magnesium, the transmission and diffusion of magnesium ion is more difficult than those of lithium ion, making electrochemical polarization and concentration polarization of magnesium particularly important (Yang et al., 2015; Chi et al., 2017; Li Q. et al., 2017; Xia et al., 2018). As a result, the overpotential of Mg anode is usually prominent. Extensive efforts have been made to the reduction of overpotential of lithium and sodium metal electrodes, and multiple methods have been proposed, including three-dimensional stable ion-conducting solid-matrix lithium metal anodes (Lin et al., 2017), composite lithium metal anode consisting of a 3D conductive scaffold with a sulfur-repellent coating (Liang et al., 2016), nitrogen, and oxygen co-doped graphitized carbon fibers with rich sodiophilic sites for sodium metal anodes (Zheng et al., 2018). Therefore, constructing a lithiopholic/sodiophilic substrate for the deposition of lithium/sodium metal have been proven to be an effective method for the overpotential reduction, which can be used as a reference for reducing the overpotential of magnesium metal batteries (Su et al., 2015; Zhang et al., 2016).

Herein, MoSe_2_ clusters consisting of two-dimensional nanosheets have been synthesized by hydrothermal method and utilized as substrate for the magnesium deposition. The as-obtained MoSe_2_ possesses a robust layered structure and is able to store magnesium within these layers (Fan et al., 2018; Lin et al., 2019). More importantly, MoSe_2_ has a magnesiopholic surface, thus facilitating the deposition of metallic magnesium on its surface. Owing to the three-dimensional porous structure and high specific surface area, the magnesiophilic MoSe_2_ electrode (MMSE) possessed large number of interfacial deposition sites of magnesium, which is beneficial to the reduction of the electrode overpotential during its operation.

## Materials and Methods

### Synthesis of MoSe_2_

First, 2 mmol of selenium powder was slowly added to 10 ml of hydrazine hydrate (85%). The mixture was continuously stirred for 1 h until it became dark red. One millimoles of Na_2_MoO_4_·2H_2_O was then dissolved in a mixed solution of 45 ml of water and 5 ml of ethylenediamine. Next, the solution (Se-N_2_H_4_·2H_2_O) was added dropwise to the Na_2_MoO_4_·2H_2_O-ethylenediamine solution and stirred for 30 min. Finally, the above mixed solution was transferred to an autoclave lined with a 100 ml polytetrafluoroethylene liner and hydrothermally treated at 200°C for 10 h. The precipitate was washed several times with deionized water and ethanol and then freeze-dried for 24 h. The samples were heat treated at 600°C for 1 h and 800°C for 1 h in an argon atmosphere to the final MoSe_2_ material.

### MMSE Preparation

MoSe_2_ (70 wt%), acetylene black (20 wt%), PVDF (10 wt%), and N-methyl-2-pyrrolidone were mixed to form a slurry and coated on Mo foil, followed by vacuum dry at 60°C for 12 h. MMSE was assembled with metal magnesium and firstly discharged at a current density of 50 μA/cm^2^ for 20 h, followed by charge and discharge at a current density of 10 A/cm^2^ and a capacity of 1 A h/cm^2^ for several hours.

### Material Characterization and Testing

The micro-morphology of molybdenum selenide was observed by field emission transmission electron microscope (TEM) (FETEM, FEI Tecnai G220, FEI NanoPorts, Ltd.) and field emission scanning electron microscope (SEM) (SU8010, Hitachi Ltd.). The powder X-ray diffractometer (D8 Advance, Bruker) and X-ray photoelectron spectroscopy (Escalab 250Xi, Thermo Fisher) were used to analyze the crystal structure of MoSe_2_. CR2025 coin-type battery was chosen for assembly test. The metal magnesium foil was used as the counter electrode, with the glass fiber as separator. The electrolyte was all perphenyl complex (APC) containing 0.2 M aluminum chloride (AlCl_3_), 0.4 M phenyl magnesium chloride (PhMgCl), and anhydrous tetrahydrofuran (THF) solvent. The constant current charge and discharge test was performed by a LAND battery analyzer.

## Results

**Figure 1:**
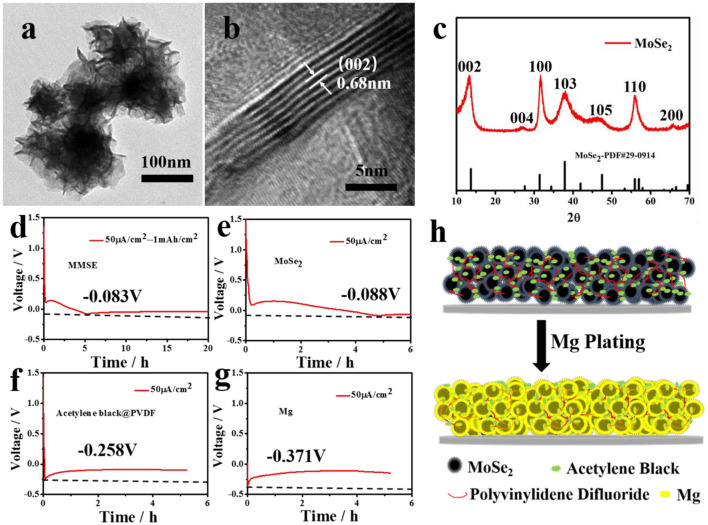
**(a)** TEM image, **(b)** HRTEM image, and **(c)** XRD spectrum of MoSe_2_. **(d–g)** Electrochemical curves showing the Mg deposition behavior on **(d)** MMSE, **(e)** MoSe_2_, **(f)** Acetylene black and PVDF, and **(g)** Mg. The current density was fixed at 50 μ A/cm^2^ for Mg plating processes. **(h)** Schematic showing the Mg deposition behavior on MMSE.

MoSe_2_ were synthesized through a hydrothermal method followed by high temperature sintering, with their morphology observed by TEM (Ge et al., 2018). As shown in Figure 1a, MoSe_2_ clearly demonstrates a cluster structure consisting of nanosheets. Ethylenediamine was added as a surfactant to control the obtained morphology so that the MoSe_2_ nanosheets self-assembled into clusters rather than arbitrarily stacked, thus tremendously increasing its contact area with the electrolyte. Such a feature was crucial for the effective current density reduction and subsequent magnesium deposition. As shown in Figure S1, the SEM image of the material also shows that it is the microscopic appearance of the nanosheet, and at the same time shows the overall morphology of the material in the micro-size (Li J. et al., 2017). The corresponding EDX also proves the uniformity of the material through element distribution (Ge et al., 2018). High-resolution transmission electron microscopy (HRTEM) in Figure 1b clearly shows the lattice fringes of MoSe_2_, with a 0.68 nm interplanar spacing corresponding to the MoSe_2_ (002) plane (Li J. et al., 2017). The X-ray diffraction (XRD) pattern of molybdenum selenide is shown in Figure 1c, which confirms the high crystallinity of MoSe_2_. The well-defined peaks at 13.5°, 31.4°, 37.9°, and 57.0° correspond to (002), (100), (103), and (110) crystal planes of MoSe_2_ (JCPDS No. 29-0914), respectively (Zheng et al., 2017). At the same time, the XPS test was performed on the material, as shown in Figure S3, and peak separation was performed on Mo 3d and Se 3d to prove the existence of Mo^4+^ and Se^2−^ respectively (Tang et al., 2014; Liu et al., 2015; Xie et al., 2016; Ding et al., 2017), proving the existence of MoSe_2_ by means of element chemical bonds.

In order to confirm the magnesiophilic feature of MoSe_2_, magnesium nucleation potentials of various materials, including MMSE, MoSe_2_, acetylene black, and polyvinylidene fluoride (PVDF), and Mg metal, were obtained from the respective discharge curves by depositing magnesium at low currents (Chen et al., 2019). At a current density of 50 μA/cm^2^, Mg plating started when the MMSE was discharged to −0.083 V (Figure 1d). The MMSE contained two parts: pure MoSe_2_, and acetylene black and PVDF. Therefore, electrochemical deposition tests were performed to investigate the magnesium nucleation potential of each part at the same current density. The pure MoSe_2_ electrode started Mg plating when discharged to −0.088 V (Figure 1e), while the acetylene black and PVDF and Mg electrodes started Mg plating when discharged to −0.258 and −0.371 V, respectively (Figures 1f,g). Therefore, the nucleation potential of pure MoSe_2_ electrode is much lower than those of acetylene black and PVDF and Mg electrodes, confirming that MoSe_2_ has a more magnesiophilic interface. The schematic diagram of magnesium deposition on MMSE is shown in Figure 1h. Cluster-like flaky MoSe_2_ preferentially embeds part of magnesium. It is reported that MoSe_2_ and carbon composite material has extremely low capacity as an electrode material for magnesium batteries (Fan et al., 2018; Lin et al., 2020). Herein, choosing this special shape of MoSe_2_ as the electrode material to assemble the magnesium battery for test, referring to Figure S2, the data displays that the amount of magnesium embedded in MoSe_2_ is low, which means the volume expansion is small, so its limited capacity determines that the amount of magnesium embedded has little effect on its microstructure and magnesiophilic surface. Therefore, the magnesiophilic surface and 3D porous strucuture of MMSE promoted the preferential deposition of magnesium and reduced the effective current density, thus being beneficial to the reduction of magnesium deposition potential.

**Figure 2:**
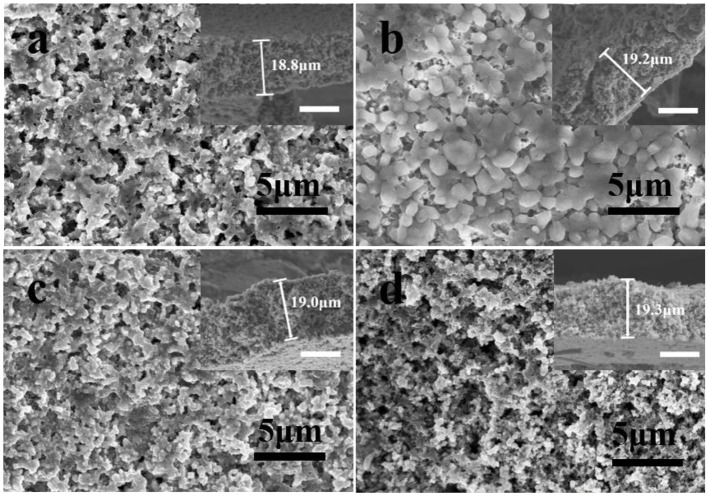
SEM images and corresponding cross-section (inset) of **(a)** pristine MMSE, MMSE after **(b)** plating 10 mA h/cm^2^, then **(c)** stripping 10 mA h/cm^2^ of Mg, and **(d)** MMSE after 1,000 cycles under a capacity of 1 mA h/cm^2^ at a current density of 10 mA/cm^2^. The white scale bar in the inset is 10 μm. Electrochemical performance of MMSE symmetric cell.

The SEM images in Figures 2a–d depict the morphologies of MMSE at different stages of plating and striping process of Mg at a current density of 10 mA/cm^2^. The 3D porous feature of MMSE can be confirmed in Figure 2a and its inset, which correspond to the pristine MMSE prior to Mg deposition. After the deposition of a large capacity of 10 mA h/cm^2^ magnesium on MMSE (Figure 2b), owing to its porous nature, no crack was generated in MMSE and the abundant pores within MMSE were filled with Mg metal. Figure 2c shows the morphology of MMSE after 10 mA h/cm^2^ of magnesium was completely released, with its morphology being very similar to those of pristine electrode shown in Figure 2a. The morphology of MMSE after 1,000 cycles under a capacity of 1 mA h/cm^2^ is shown in Figure 2d. The MMSE after 1,000 cycles shows similar morphology with that of pristine MMSE (Song et al., 2019). The insets at the upper right corner of each SEM image are the cross-sectional view of corresponding electrode, with similar cross-sectional thicknesses, being 18.8, 19.2, 19.0, and 19.3 μm, for pristine MMSE, MMSE after first Mg deposition then first Mg exfoliation, and MMSE after 1,000 cycles, respectively (Chen et al., 2019). The stable electrode structure of MMSE after deposition of large amounts of Mg was benefited by the three-dimensional porous structure and large magnesiophilic interface of MMSE.

**Figure 3:**
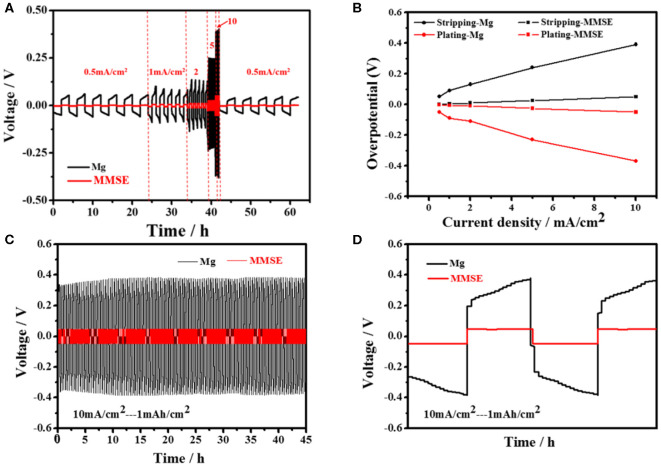
**(A)** Voltage profile of Mg symmetric cell (black) and that of MMSE symmetric cell (red) at different rate various from 0.5 to 10 mA/cm^2^. **(B)** Overpotentials for plating and stripping of Mg electrode and MMSE during cycling. **(C)** The voltage profiles of Mg symmetric cells (black) and MMSE symmetric cells (red) at the current densities of 10 mA/cm^2^ and **(D)** magnified Mg plating/stripping profiles.

To investigate its constant current electrochemical performance, symmetrical cells of MMSE were assembled to evaluate the voltage change during Mg plating/stripping. Comparing the deposition/stripping curves between MMSE and Mg electrodes at different current densities shown in Figure 3A, it can be clearly observed that the deposition/stripping potentials of MMSE are much smaller than those of Mg electrode. The deposition/stripping potentials of the MMSE and Mg electrodes in Figure 3A were extracted and plotted against various current densities in Figure 3B. Specifically, at the current density of 0.5, 1, 2, 5, 10 mA/cm^2^, the magnesium deposition potential of MMSE and Mg are −0.003, −0.005, −0.011, −0.026, −0.051 V, and −0.05, −0.09, −0.11, −0.23, −0.37 V, respectively, confirming the much smaller hysteresis voltages of MMSE than those of metallic magnesium. In addition, for more detailed data comparison with other methods in reducing the overpotential of magnesium metal anode, Li et al. added a small amount of iodine to the (TFSI) _2_-DME electrolyte as an additive (Li et al., 2018). Voltage hysteresis is defined as the voltage difference between magnesium plating and stripping, which depends on multiple factors, including current density, charge transfer resistance, and interface characteristics. MMSE, with a large magnesiophilic interface, can effectively reduce both equivalent current density and Mg nucleation potential, thus delivering low overpotentials at various current densities. In the long cycling process under a current density of 10 mA/cm^2^ (Figure 3C), both the MMSE and Mg electrode show stable cycles. But the voltage lag of the MMSE is much smaller than that of the Mg electrode. The much larger overpotential of Mg electrode can be clearly observed in the locally enlarged cycling curve in Figure 3D. Besides, owing to the concentration polarization of magnesium ions by the solvation effect, it can be observed that the deposition/stripping potential of Mg gradually increased in each cycle (Canepa et al., 2015). Referring to Figure S4 for comparison of other current densities, under a current density of 1 and 5 mA/cm^2^, the same as 10 mA/cm^2^, MMSE does have better performance in reducing overpotential than magnesium metal electrode. In contrast, the potential of the MMSE remained stable during the plating/striping process.

## Discussion

In summary, MMSE has been successfully fabricated by utilizing magnesiophilic flaky MoSe_2_ nanoclusters synthesized by hydrothermal method and heat treatment. The magnesiophilic interface of MMSE has been confirmed by magnesium deposition electrochemical tests. Benefiting from its 3D porous structure, MMSE shows excellent structural stability after deposition and stripping of large-capacity of magnesium. By assembling in a symmetric battery, MMSE shows much smaller overpotential of that of metal magnesium under different current densities, which shows the excellent ability of MMSE in reducing the hysteresis voltage of magnesium anode. This work shows a method to reduce the Mg overpotential by adopting a three-dimensional MoSe_2_-based electrode with magnesium-affinitive interface, which provides an alternative route for the development of Mg metal anode.

## Data Availability Statement

The raw data supporting the conclusions of this article will be made available by the authors, without undue reservation.

## Author Contributions

All authors listed have made a substantial, direct and intellectual contribution to the work, and approved it for publication.

## Conflict of Interest

The authors declare that the research was conducted in the absence of any commercial or financial relationships that could be construed as a potential conflict of interest.
