# Supplementary material for: Magnesiophilic Interface of 3D MoSe2 for Reduced Mg Anode Overpotential
Source: Front Chem. 2020 Jun 18;8:459. doi: 10.3389/fchem.2020.00459 (PMC7314988; doi:10.3389/fchem.2020.00459)
Supplement: Supplementary file 1 [file Table_1.DOCX]

Supporting Information

**Magnesiophilic Interface of 3D MoSe_2_ for Reduced Mg Anode Overpotential**

Tong Shen^1^, Chengzhao Luo^1^, Yu Hao^1^ and Yu Chen^1, 2^*

1 School of Optoelectronic Science and Engineering & Collaborative Innovation Center of Suzhou Nano Science and Technology, Soochow University, Suzhou, China

2 National University of Singapore Suzhou Research Institute, Dushu Lake Science and Education Innovation District, Suzhou 215123, P. R. China

*** Correspondence:**

Yu Chen, chenyu_ny@suda.edu.cn


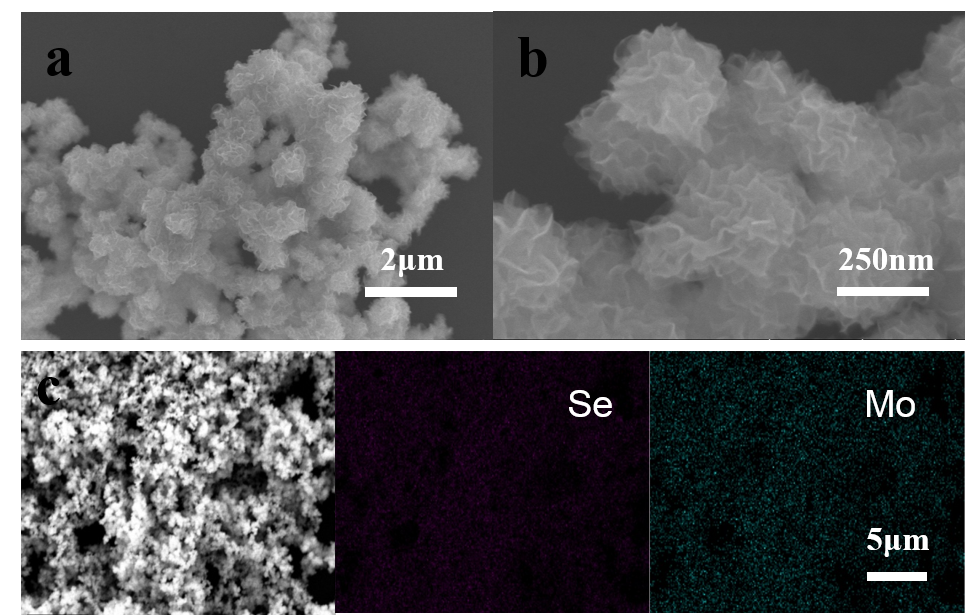


**Figure S1**. (a-b) SEM image, and (c) EDX image of MoSe_2_


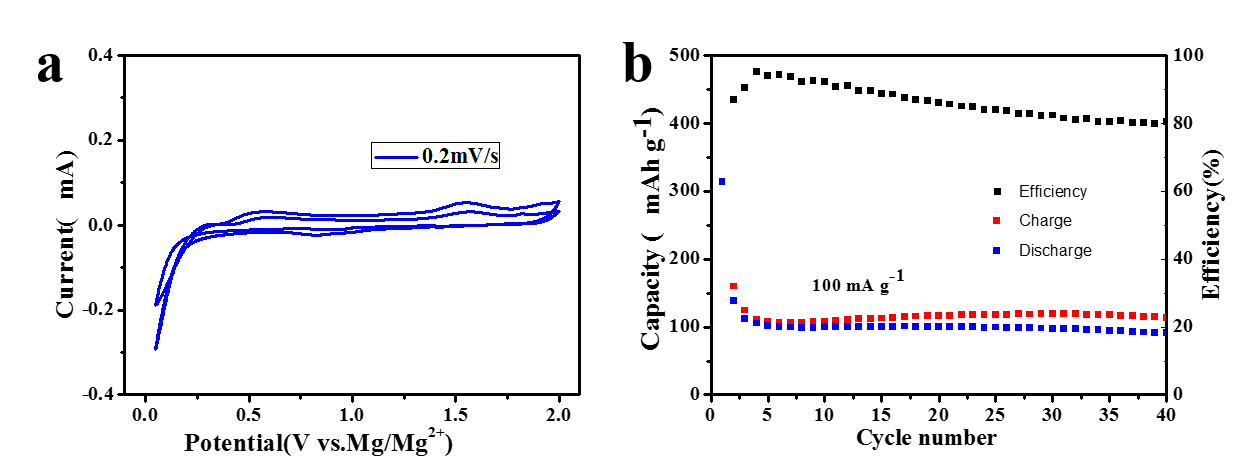


**Figure S2**. (a) CV curves and (b) discharge-charge cycling curve of MoSe_2_.


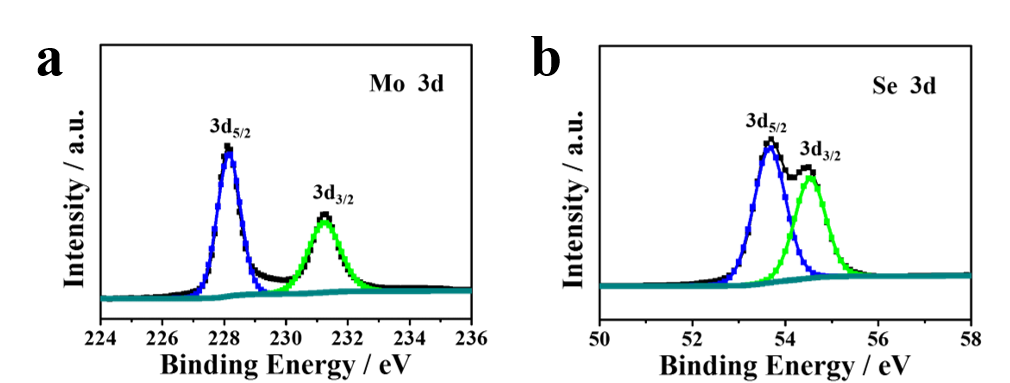


**Figure S3**. XPS spectra of (a) Mo 3d and (b) Se 3d.


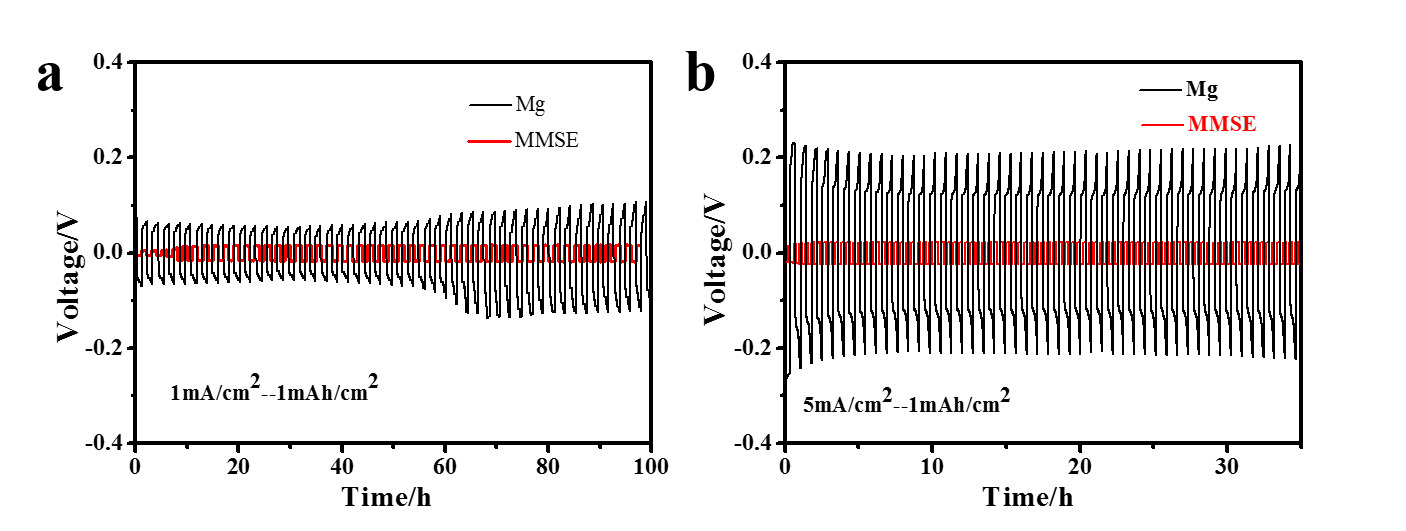


**Figure S4**. The voltage profiles of Mg symmetric cells (black) and MMSE symmetric cells (red) at the current densities of (a) 1 mA/cm^2^ and (b) 5 mA/cm^2^.
